# Phytosynthesis of Silver Nanoparticles Using *Leonurus cardiaca* L. Extracts

**DOI:** 10.3390/ma16093472

**Published:** 2023-04-29

**Authors:** Ioana Catalina Fierascu, Irina Fierascu, Anda Maria Baroi, Camelia Ungureanu, Simona Spinu, Sorin Marius Avramescu, Raluca Somoghi, Radu Claudiu Fierascu, Cristina Elena Dinu-Parvu

**Affiliations:** 1 Faculty of Pharmacy, University of Medicine and Pharmacy “Carol Davila”, 37 Dionisie Lupu Str., 030167 Bucharest, Romania; ioana.fierascu@drd.umfcd.ro (I.C.F.); cristina.dinu@umfcd.ro (C.E.D.-P.); 2 National Institute for Research & Development in Chemistry and Petrochemistry—ICECHIM Bucharest, 202 Spl. Independentei, 060021 Bucharest, Romania; irina.fierascu@icechim.ro (I.F.); anda.baroi@icechim.ro (A.M.B.); raluca.somoghi@icechim.ro (R.S.); 3 Faculty of Horticulture, University of Agronomic Sciences and Veterinary Medicine of Bucharest, 011464 Bucharest, Romania; simonaspinu91@gmail.com; 4 Faculty of Chemical Engineering and Biotechnologies, University “Politehnica” of Bucharest, 313 Splaiul Independentei Str., 060042 Bucharest, Romania; ungureanucamelia@gmail.com; 5 Faculty of Chemistry, University of Bucharest, 91–95 Splaiul Independentei, 050095 Bucharest, Romania; sorin_avramescu@yahoo.com

**Keywords:** phytosynthesis, *Leonurus cardiaca* L., silver nanoparticles, antioxidant, antimicrobial

## Abstract

The present work describes, for the first time in the literature, the phytosynthesis of silver nanoparticles using *Leonurus cardiaca* L. extracts. The influence of the extraction method (classical temperature extraction and microwave extraction), as well as of the extract concentration on the characteristics of the nanoparticles, was studied using analytical methods, such as UV-Vis spectrometry, X-ray diffraction, dynamic light scattering, and transmission electron microscopy. Experimental data suggest that use of lower extract concentration leads to smaller dimensions nanoparticles, the same effect using the extract obtained by microwave-assisted extraction. The smallest recorded crystallite sizes (by X-ray diffraction) were under 3 nm. The antioxidant properties (determined by the DPPH assay) and the antimicrobial potential (determined against Gram-negative and Gram-positive strains) are enhanced by the phytosynthesis process (as demonstrated by the comparison of the nanoparticles’ properties with the parent extracts). The present work could also represent an important step in obtaining nanoparticles with enhanced properties and controlled morphologies, but also offers information on the phytosynthesis of metallic nanoparticles using low extract concentrations.

## 1. Introduction

The amazing world of plants offers new possibilities to obtain specific materials with a vast range of potential applications. Nowadays, plant-derived materials are used in various areas, from novel therapeutic instruments for disease treatment to environmental applications or development of sensory materials [1,2,3]. Due to their rich chemical composition, plants are considered to be potential candidates for the development of a new generation of nanomaterials, using “eco-friendly” methods, with a much lower environmental impact, involving the use of non-hazardous reagents and chemicals, as well as having lower production cost and energy consumption [4,5]. Even if we are speaking of medicinal and aromatic plants, non-edible plants or agro-wastes, there is great interest in the phytosynthesis of metal nanoparticles using such plants extracts; the main challenge in the phytosynthesis process is represented by the development of nanoparticles (NPs) with controlled compositions, sizes, shapes, and polydispersity, using different types of plant extracts [6]. As in any nanoparticles’ synthesis process, one important step in the development of a viable nanoparticles solution is represented by the NPs stabilization, in order to prevent their agglomeration or oxidation; the various phytoconstituents present in the natural extracts have the role of both reducing and capping agents, thus the composition of the extracts has a very important role in the final dimensions and morphologies of the developed NPs [7].

During the present nanotechnological area, metallic or metal oxides nanoparticles find extensive application in different areas, from advanced sensors [8] to environmental protection [9,10], and from the biomedical area [11] to day-by-day products [12,13]. As their wide range of application grew, so did the various concerns regarding their potential detrimental effects on the environment and human health [14]. Among the *green* synthesis methods, of particular interest is the phytosynthesis process (defined as the development of nanomaterials using plant extracts) [15,16].

Recent studies presented phytosynthesis of nanoparticles, such as noble metals, copper, zinc, iron, etc., with potential anticancer, antibacterial, antioxidant, anti-inflammatory, and antiviral properties, thus proposing them as good candidates in medical therapies [17,18]. The main advantage of phytosynthesized nanoparticles is, for this type of application, the contribution of the phytoconstituents to the final biomedical properties [15]. However, the potential of such nanoparticles exceeds the bio-medical area. Nanoparticles developed using plant extracts were proven to have potential applications in various other industries, such as, i.e., sensors industry, environmental protection [19], optoelectronics [20], or as biostimulants and plant protection agents [21,22]. 

Known as the motherwort, belonging to the genus *Leonurus* (family Lamiaceae), *Leonurus cardiaca* L. is a perennial plant growing up to 1 m tall, with aerial stems growing from rhizomes and lobed leaves covered with bristles; it has pink flowers, grouped in clusters in the leaf axils [23]; the plant is often used in folk medicine across Asia, Europe, and America [24,25]. More recently, motherwort was demonstrated in several scientific studies to possess cardioprotective [26], immunomodulatory [27], antimicrobial [28], antioxidant, or antidepressive [29] effects, among others.

All these properties, and many others, are in strong correlation with the composition of the plant. Generally speaking, the composition of motherwort is dominated by furan diterpenes, alkaloids, sterol, iridoids, flavonoids, and minerals [30]; literature data also confirmed the presence of chlorinated iridoid glucoside [31], ferulic acid, chlorogenic acid, caffeic acid, rutoside, lavandulifolioside, verbascoside, isoquercitrin and stachydrin [32,33], caryophyllene, α-humulene, α-pinene, pinene, linalool or limonene [34].

Up to date, another species from the *Leonurus* genus (*Leonurus japonicus* Houtt.) was proven to be successfully applied for the phytosynthesis of silver nanoparticles with enhanced antibacterial properties [35]. In this context, we propose the phytosynthesis of silver nanoparticles (AgNPs) using extracts obtained from native of *L. cardiaca* aerial parts (as a source of bioactive compounds).

Besides the complex biological activity of the plant itself, its rich composition in phytoconstituents recommends this plant for the synthesis of metallic nanoparticle with antimicrobial properties [36]. One of the main reasons for developing the present study is represented by the lack of any scientific data on this topic, this being, up to our knowledge, the first study demonstrating the use of *L. cardiaca* extracts for the phytosynthesis of noble metal nanoparticles and presenting their characterization. The current study details the obtaining of the natural extracts, their analytical characterization, as well as the influence of the extract concentration on the characteristics of the obtained nanoparticles.

## 2. Materials and Methods

### 2.1. Vegetal Material

**Figure 1:**
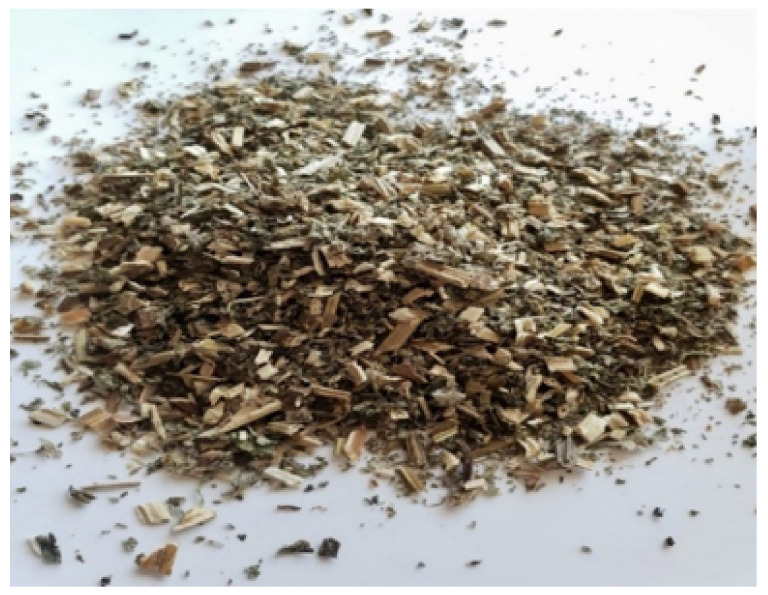
Dried and processed aerial parts of *L. cardiaca* used in the experiments.

The vegetal material (aerial parts of *L. cardiaca*) was collected in the Bucharest area, Romania, from plants grown from certified seeds (Figure 1). 

The drying of the plant material was performed away from the direct sun rays, until constant mass was achieved, a method previously proven to preserve the active principles during the drying process [37]. After drying, the vegetal material was grounded with a GRINDOMIX GM 200 mill (Retsch GmbH, Haan, Germany) and kept in airtight containers.

### 2.2. Obtaining of Natural Extracts 

In order to evaluate the influence of the extraction method on the characteristics of the NPs, two extraction methods were selected, considering both the efficiency of the procedures and the ease in operation [38].

The first extraction procedure selected was the *classical temperature extraction;* for this method, the vegetable material was mixed with the solvent (ethanol: water = 1:1), and the extraction was performed using a UN 110 oven (Memmert GmbH, Schwabach, Germany), in the following conditions: extraction time 3 h, extraction temperature 70 °C. The obtained extract was encoded L_t_.

The second extraction method selected was the *microwave-assisted extraction*; the vegetal material and solvent mixture (ethanol: water = 1:1) was subjected to microwave heating using an Ethos Easy Advanced Microwave Digestion System (Milestone Srl, Sorisole, Italy), under the following parameters: extraction time 30 min., extraction temperature 70 °C, microwave power 800 W. The extract was encoded L_mw_. In both cases, the ratio vegetal material/solvent was 1/12 (*w*/*v*); after natural cooling to room temperature, the extracts were filtered using filter paper and reduced using a Laborota 4000 rotary evaporator (Heidolph Instruments, Schwabach, Germany). The extracts were further lyophilized (using a Christ LSC Alpha 2–4-LSC lyophilizer, Martin Christ Gefriertrocknungsanlagen GmbH, Osterode, Germany), and the obtained powders were stored for further use. 

The ethanol used as solvent was reagent quality (Chimreactiv, Bucharest, Romania), while the bidistilled water was obtained in the laboratory using a GFL 2102 water still (GFL, Burgwedel, Germany).

### 2.3. Characterisation of Natural Extracts 

The composition of the extracts was evaluated using phytochemical assays (total content of phenolic compounds, respectively total content of flavonoids), as well as high-precision liquid chromatography (HPLC), for the quantification of selected compounds. All reagents were used as received, without further purification.

The results of the phytochemical assay (methods being previously exhaustively presented by our group [39]) are expressed as milligrams of gallic acid equivalents (GAE, for the total phenolics content, gallic acid provided by Merck KgaA, Darmstadt, Germany), respectively as milligrams of rutin equivalents (RE, for total flavonoids content, rutin provided by Merck KgaA, Darmstadt, Germany). For each assay, five determinations were performed, the results representing the average of the determinations ± the standard error of the mean.

High-Performance Liquid Chromatography analyses were performed using a chromatograph equipped with a diode-array detector (HPLC-DAD, Rigol Technologies Inc., Beijing, China), using a method previously described [39]. The compounds selected for quantification using HPLC consisted of phenolic and hydroxycinnamic acids (gallic acid, chlorogenic acid, coumaric acid, caffeic acid), flavonoids (catechin, hyperoside, rutin, naringin, naringenin, epicatechin, genistein), and anthocyanidins (malvidin, delphinidin). For each compound, a five-point calibration curve was constructed (10–400 µg/mL), using standard materials (Merck KgaA, Darmstadt, Germany).

For compositional evaluation, the lyophilized extracts were diluted with bidistilled water to reach a final concentration of 5 mg/mL.

### 2.4. Phytosynthesis and Characterization of Silver Nanoparticles 

For the NPs phytosynthesis, the dried extracts were redissolved in bidistilled water at five concentrations (5, 2.5, 2, 1.25, respectively 1 mg/mL); the resulting solutions were mixed with the metallic salt precursor (10^−3^ M silver nitrate solution, Chimreactiv, Bucharest, Romania), in equal volumes, under natural light, at room temperature. No stirring was applied to the mixture. The encodings of the obtained samples are presented in Table 1.

The development of the NPs was monitored using the following analytical techniques:

- UV-Vis spectrometry, for the evaluation of the phytosynthesis process (as AgNP usually exhibit an absorption maximum in the 400–550 nm region), to estimate the NPs dimensions, and to evaluate the stability of the NPs solutions (by recording the UV-Vis spectra of the NPs solution after 9 months from the synthesis) [38]. For the determinations, a Rigol Ultra 3660 UV-Vis spectrometer (Rigol Technologies Inc., Beijing, China, optical resolution 0.5 nm) was used, the analyses being performed in the range 375–550 nm.
- Dynamic light scattering (DLS), for the determination of the NPs’ hydrodynamic diameter [40]; for DLS analysis, five/ten determinations were performed for each sample, using a Zetasizer Nano ZS ZEN 3600 instrument (Malvern Instruments, Malvern, UK) capable of determining particle size in the range 0.6–6000 nm.
- X-ray diffraction (XRD), for the determination of the crystallin structure and crystallite size [41], using a 9 kW Rigaku SmartLab diffractometer (Rigaku Corp., Tokyo, Japan), under the following operating parameters: 45 kV, 200 mA, Cu_Kα_ radiation (1.54059 Å), 2θ/θ scanning mode; the diffractograms were recorded between 7 and 90° (2θ). Components were identified by comparison with ICDD data (ICDD, Newtown Square, PA, USA), using the equipment’s software (PDXL, v. 2.7.2.0, Rigaku Corp., Tokyo, Japan), while the crystallite size was determined using the Debye–Scherrer Equation (1):
  (1) $$Dp=\frac{\left(K\times \lambda \right)}{\left(\beta \times cos\theta \right)}$$
  where *Dp* = the average size of the crystallites, *K*—the Scherrer constant (*K* = 0.94), *β* = the width at half-height of the diffraction maximum, *θ* = the Bragg angle, *λ* = the wavelength—1.54059 Å).
- Transmission Electron Microscopy (TEM) for the visualization of selected NPs, using a Tecnai G2 F20 TWIN Cryo-TEM equipment (FEI Company, OR, USA), at an accelerating voltage of 300 kV and a resolution of 1 Å. The microscope was equipped with an energy dispersive X-ray spectroscopy (EDX) accessory (Oxford Instruments, Abingdon, UK), allowing the evaluation of the elemental composition of the samples.

### 2.5. Evaluation of Biological Properties 

The antioxidant properties of both the extracts and the nanoparticles solutions were determined using the DPPH (2,2-diphenyl-1-picrylhydrazyl) assay, by measuring the discoloration of the stable free radical solution (Sigma Aldrich, St. Louis, MO, USA), as previously exhaustively presented by our group [39,42]. The determination was performed in five replicates, the results being presented as the average of the determinations ± the standard error of the mean.

The antibacterial testing of the s”mple’ studied in this manuscript was carried out on two bacterial strains *Escherichia coli* (Gram (-) bacterium) and *Enterococcus faecalis* (Gram (+) bacterium). These microorganisms were chosen because of their pathogenicity.

*Enterococcus faecalis* can be spread from person to person. Many countries have adopted strict regulations on tap and bottled water, as well as bathing water, in order to avoid the contamination with *E. faecalis* [43]. This bacterium can become pathogenic for immunocompromised people or those who already have a chronic pathology such as diabetes. When their numbers increase significantly, enterococci produce proteases, allowing them to break down the normal barriers between intestinal tissue and blood. They can then invade the bloodstream and cause various infections, such as septicemia, urinary tract infections, bacteremia, meningitis, endocarditis, peritonitis, or intra-abdominal abscesses. In addition to immunosuppression, medical equipment as well as medical personnel are at risk; another potential risk is represented by the excessive intake of antibiotics, which can promote the emergence of enterococci [44,45]. *Enterococcus faecalis* is commonly found in enterococcal infections (80–90%), and its increasing resistance to antibiotics has made it a bacterium of concern, as nosocomial infections are a real health problem. *E. faecalis* has a natural resistance to certain antibiotics, such as cephalosporins, and is tolerant to penicillin. Several studies [46,47] show that it is sensitive, among others, to gentamicin. For this reason, in the case of the antibacterial assay, gentamicin sulfate was chosen as a positive control.

Some types of *Escherichia coli* (Shiga toxin *E. coli*, abbreviated STEC) produce a powerful toxin known as Shiga toxin, which can cause fatal infections [48]. Other infections can lead to kidney failure, especially in children or the elderly. This bacterium can cause urinary, intestinal, and even respiratory infections [49]. Nosocomial infections represent another important aspect of the infectivity of this bacterium, being a characteristic of hospital endemic multiresistant *E. coli* strains [50].

All the antimicrobial determinations were performed in triplicate. Distilled water was used as a negative control and gentamicin sulfate (Sigma Aldrich, Saint Louis, MO, USA) at a concentration of 10 µg/mL was used as a positive control. All bacteria strains were grown in Luria Bertani Agar (Miller, LBA) plates incubated at 37 °C. The antibacterial assay was performed by the agar well diffusion method [42]. Briefly, sterile culture medium was poured in Petri dishes and 1 mL of bacteria strains was spread on the LBA plates surface. Wells with a diameter of 6 mm were made with the help of a sterile borer, and 50 µL of the samples were added in each well; afterwards, the Petri dishes were placed in the incubator at 37 °C for 18–24 h. The qualitative determination of the antibacterial effect was achieved by measuring the zone of inhibition (ZOI, mm) according to the method described by Ponce et al. [51]. 

### 2.6. Statistical Analysis and Data Representation 

The results obtained after multiple parallel determinations (detailed for each method) were analyzed for statistical significance using analysis of variance (one-way ANOVA) and the Tukey test to determine significant differences between means. Significant differences were set at *p* ≤ 0.05. Results shown for the determinations represent mean ± standard error of the mean (SE) of independent determinations. OriginPro 2018 Data Analysis and Graphing Software v. 95E (OriginLab Corporation, Northampton, MA, USA) was used for all the graphic representation.

## 3. Results

### 3.1. Extracts Compositional Evaluation

As previously mentioned, the NPs characteristics are in strong dependency with the composition of the extracts used for phytosynthesis. In order to obtain an image on this parameter, the general composition of the extracts was evaluated by means of total phenolic content and total flavonoids content, while the selected components were quantified by HPLC (results presented in Table 2).

By comparing the results of the phytochemical assays with the literature data [52,53,54] regarding *L. cardiaca* extracts, it can be observed that our results are generally lower than the ones previously reported (results of literature data ranging from 7pprox.. 2 to 200 mg GAE/g). However, the results are similar to those obtained by Armatu et al. [53] (2 mg GAE/g), presented in a comparable study, as it deals with vegetal material harvested from similar geographical conditions. By comparison with the same literature data [52], the total flavonoids content is significantly higher in the current study (7pprox.. 2.5-times higher flavonoids content than the value reported in the literature); however, due to the different ratios vegetal material/solvent (in the cited work, the authors used a ratio 1:30 *w*/*v*), the two results are similar. 

The differences recorded could be e”plained, besides the influence of local environmental factors, by the value of the cultivar and the particular conditions on harvesting time, as well as by the mild extraction conditions and the solvents used in the present study, which could negatively influence the amount of extracted phytoconstituents. However, we can conclude that the motherwort grown in native conditions presents a lower intake of active constituents, especially when speaking of total phenolics, and at the same time, a higher intake of total flavonoids; this is a very interesting observation, as our group also presented the same conclusion regarding the composition of *Echinacea purpurea* L. flowers [39]. 

Comparing the two extraction methods, it can be observed that the classical temperature extraction leads to higher total phenolics content, compared with the microwave extraction, while the difference between the two methods, in terms of total flavonoids, is not statistically significant. Regarding these differences, it is known that the value of TPC, due to the presence of a hydroxyl group, is dependent on the solvent’s polarity [55]. In our case, as the same solvent was used for both methods, the recorded difference could be assigned to the extraction method used. As also observed by Upadhya et al. [56] in a study on *Achyranthes aspera* L., the value of TPC is dependent on two parameters that were varied in the present study: extraction method and extraction time. As the cited authors observed a higher value for the microwave extraction, compared with ultrasound extraction and continuous shaking extraction, most probably in our case, the difference between the TPC extraction efficiency of the two methods is counterbalanced by the influence of the extraction time (30 min for microwave extraction, respectively 3 h for temperature extraction). As such, most probably, the extraction time using microwave-assisted extraction should be increased in order to reach higher TPC values. However, this hypothesis should be thoroughly verified in future studies, as the extraction parameters are unique for each vegetal material. Regarding the total flavonoid content, also influenced by the extraction method and extraction time as demonstrated by Nurcholis et al. [57], we could assume that the lack of significant differences between the results obtained in the current study signifies that proper extraction parameters were achieved.

For the discussions regarding the HPLC quantification of the selected compounds, the same literature data previously cited will be used. By comparing our results with the ones presented by Angeloni et al. [52], it can be observed that the present work reports lower concentrations (although comparable) of chlorogenic acid (2.2–2.8 mg/g reported in [52]), caffeic acid (7pprox.. 0.7 mg/g), and rutin (7pprox.. 2.5 mg/g), although higher than the ones reported by Emmanuel et al. [58] (in terms of caffeic acid—value reported in the literature 7pprox.. 0.11 mg/g and gallic acid—7pprox.. 0.03 mg/g), respectively lower in terms of chlorogenic acid (value reported—7pprox.. 2 mg/g). The content in chlorogenic acid is also lower when compared with the results presented by Koshovyi et al. [59] in motherwort tincture, but higher values were recorded for caffeic acid. The value recorded for the coumaric acid is also comparable with the one reported by Meškauskaitė [60]. The lower amounts can be, to some extent, explained by the lower total phenolics content recorded by comparison with the literature data. The study of Emmanuel et al. [58] also reports the presence of several anthocyanins (water-soluble vacuolar pigments), while the present work identifies the presence of anthocyanidins (sugar-free counterpart of anthocyanins). Their presence is most probably related to the presence of flowers in the harvested aerial parts of *L. cardiaca* used in the present study. 

In terms of flavonoids, our study identified higher contents in rutin, hyperoside, and catechin, compared to the study of Koshovyi et al. [59]. Other flavonoids identified in the current work (i.e., naringin and naringenin) were not quantified in the literature, as to our knowledge.

When comparing the two extraction methods, it can be observed that the phenolic acids are usually in higher quantities in the extract obtained by temperature extraction (although the differences are not always statistically significant), with the exception of caffeic acid (significantly higher in the microwave-obtained extract). The anthocyanidins are usually in higher amounts in the extract obtained via the microwave method (malvidin being quantified only in L_mw_), while the flavonoids have an irregular distribution, with some being present in higher quantities in L_t_ (hyperoside, rutin), while others (naringin, naringenin, and, especially, catechin) being found in higher quantities in L_mw_. These results can be correlated with the phytochemical assays previously discussed.

Although the present work is not intended to elucidate the composition of the motherwort extracts, the results presented can constitute an important starting point for future studies in this area, as well as the evaluation of an important phytosynthesis process parameter. 

### 3.2. Analythical Characterization of the NPs

**Figure 2:**
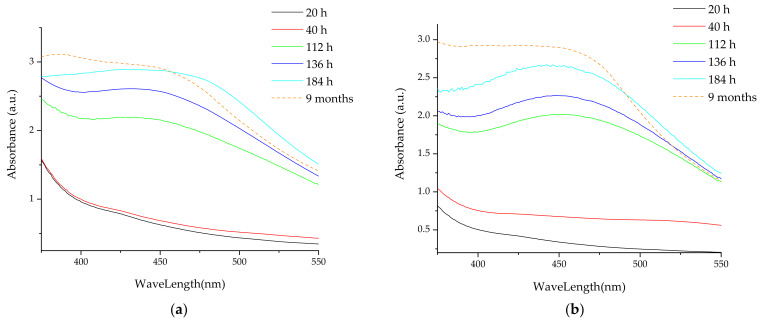

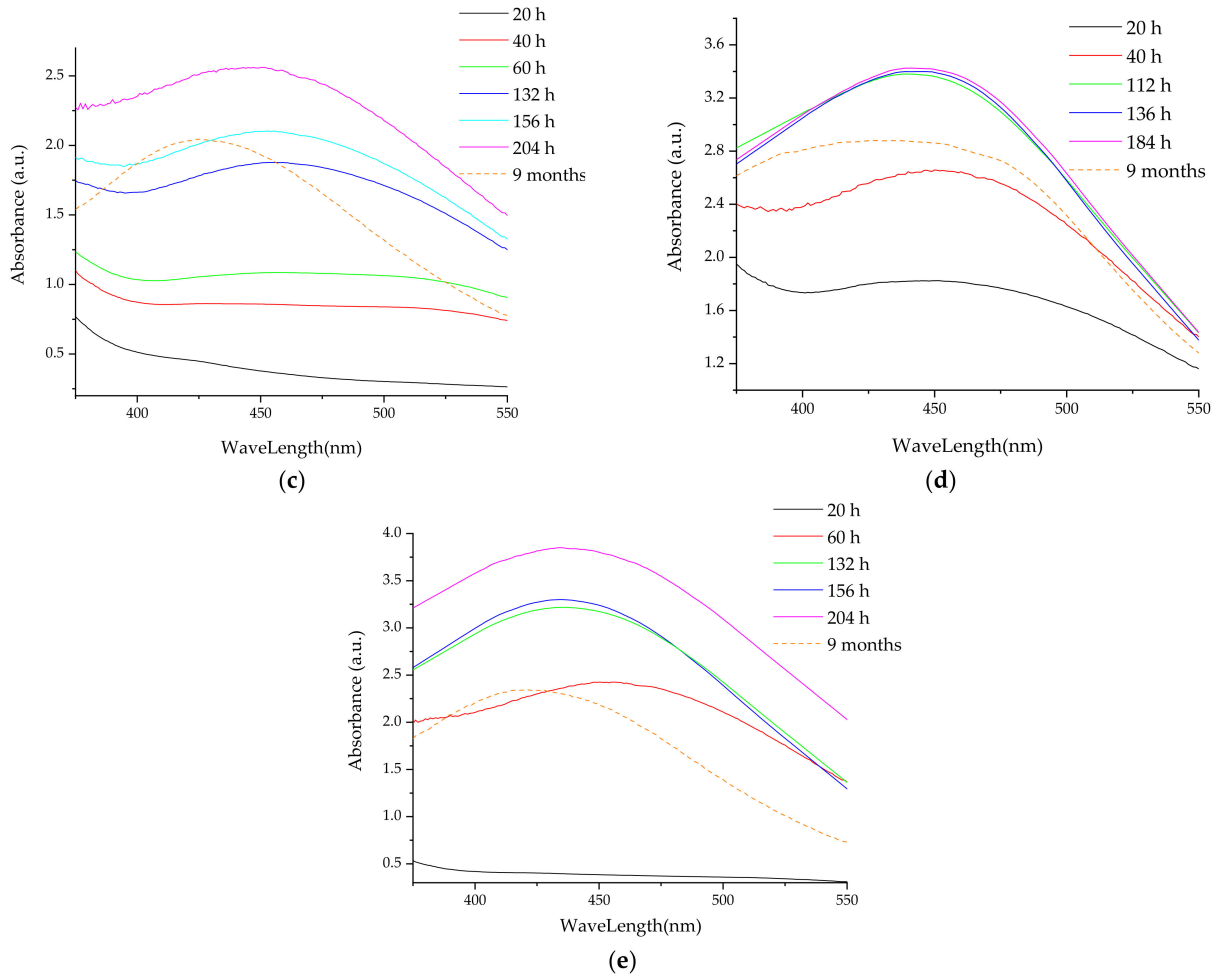
UV-Vis spectra of the developed nanoparticles using *L. cardiaca* extracts obtained by classical temperature extraction: (**a**) sample LT5; (**b**) sample LT2.5; (**c**) sample LT2; (**d**) sample LT1.25; (**e**) sample LT1.

**Figure 3:**
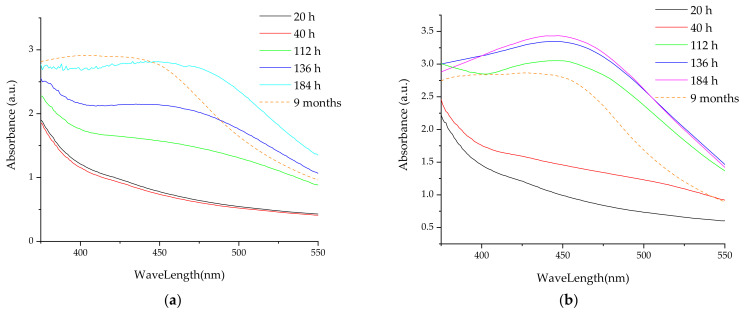

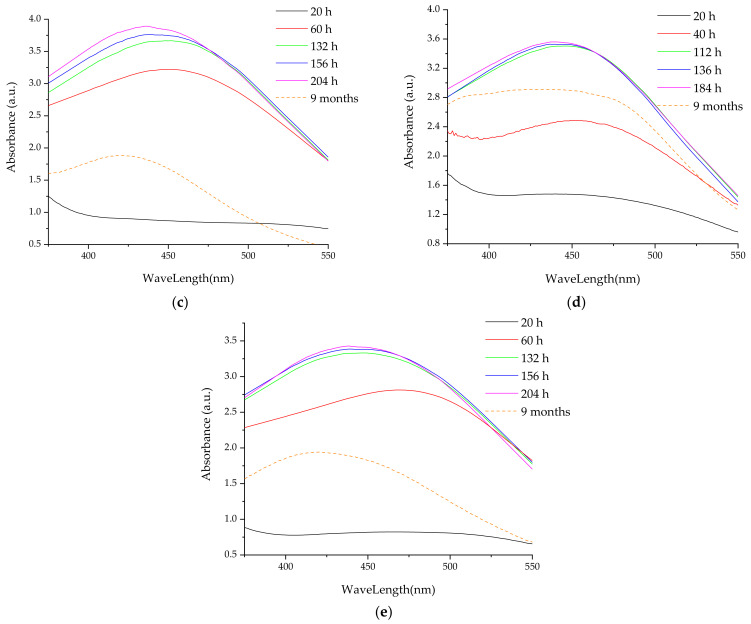
UV-Vis spectra of the nanoparticles developed using extracts obtained by the microwave-assisted extraction: (**a**) sample LM5; (**b**) sample LM2.5; (**c**) sample LM2; (**d**) sample LM1.25; (**e**) sample LM1.

As previously demonstrated by our group [38,39,42], the formation of silver nanoparticles can be monitored using UV-Vis spectrometry, due to the apparition of specific absorbance maxima around 550 nm. Figure 2 presents the UV-Vis spectra of the NPs developed using the extract obtained by classical temperature extraction (further referred to as LT series), while Figure 3 displays the UV-Vis spectra of the NPs phytosynthesized using extracts obtained by the microwave-assisted method (further referred to as LM series), with corresponding data presented in Table 3.

From Figure 2a–e, it can be observed that, in the case of motherwort extracts obtained by classical temperature extraction, the most effective concentration of extract for phytosynthesis is the concentration of 1 mg/mL. In this case, the most well-defined maximum absorption and the smallest shift from the initial maximum position are observed, even after a period of 9 months. For all the concentrations used, after a period of 9 months, a hypsochromic shift of the maxima associated with the presence of silver nanoparticles is observed, except for sample LT5, where a small bathochromic shift can be noticed, suggesting a slight agglomeration tendency in this case. In general, the smallest apparent sizes are observed for the sample with the lowest extract concentration (LE1), although the observed sizes are in a narrower range of values compared to nanoparticles phytosynthesized using *Echinacea* extracts following a similar protocol, as previously presented by our group [39].

A similar trend is observed in the case of extracts obtained using microwaves extraction. From Figure 3a–e, it can be observed that, for this extraction method, with the decrease in the extract concentration, the maximum characteristic of silver nanoparticles is more clearly defined. It is worth noticing that, for samples LM1.25 and LM1 (containing the lowest concentrations of extract), phytosynthesis is evident as early as 20 h after the beginning of the process. In addition, although for the first determinations (at 20 h, respectively at 40/60 h), the evaluated particle dimensions in the case of LM1 are larger, at the end of the monitored period (9 months), the particle dimensions for LM1 are smaller. As also observed for the phytosynthesis process using extracts obtained by the classical temperature extraction, the relative dimensions of the nanoparticles are in the range of 31–45 nm; for both cases, the particle dimensions are smaller than those obtained by our group using *Echinacea* extracts [39]. In conclusion, both for relatively short evaluation times (184/204 h) and at the end of the stability evaluation period (9 months), a good stability of the nanoparticles can be observed, with no tendency of aggregation (which could be suggested by a bathochromic shift), with one single exception. 

**Figure 4:**
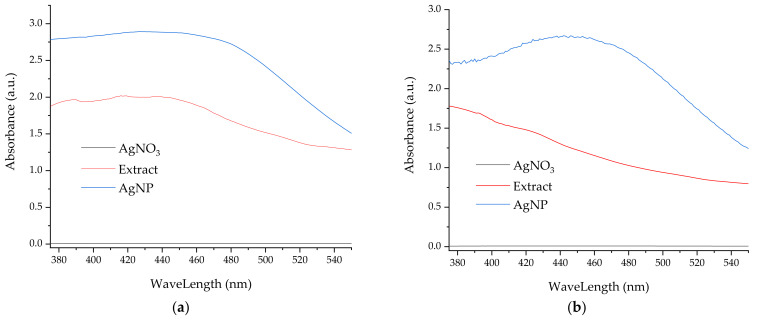

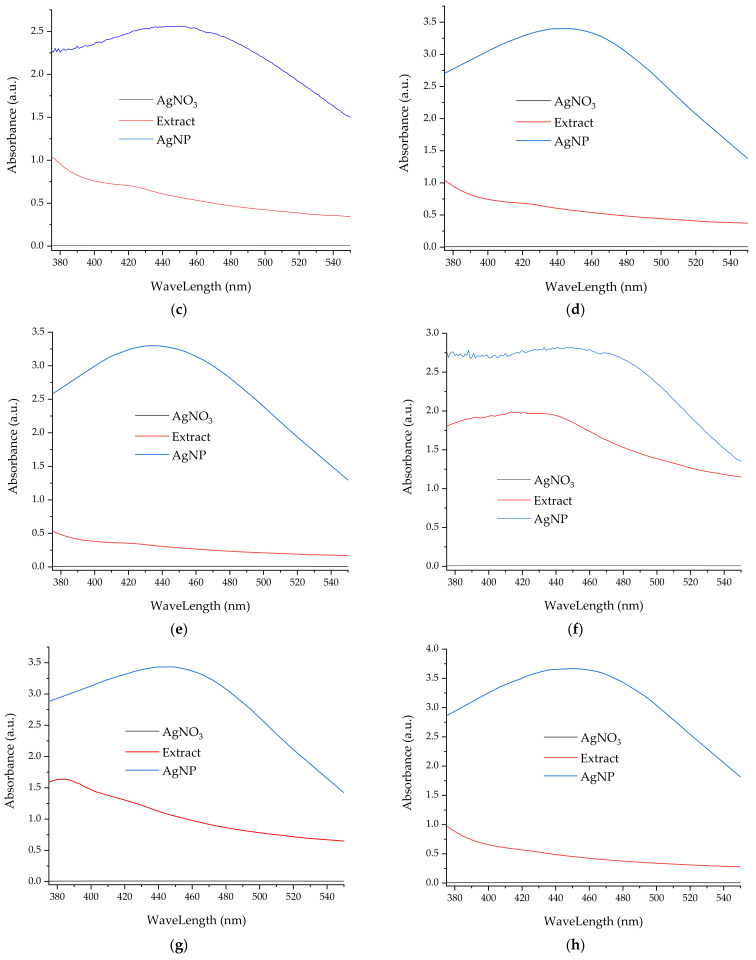

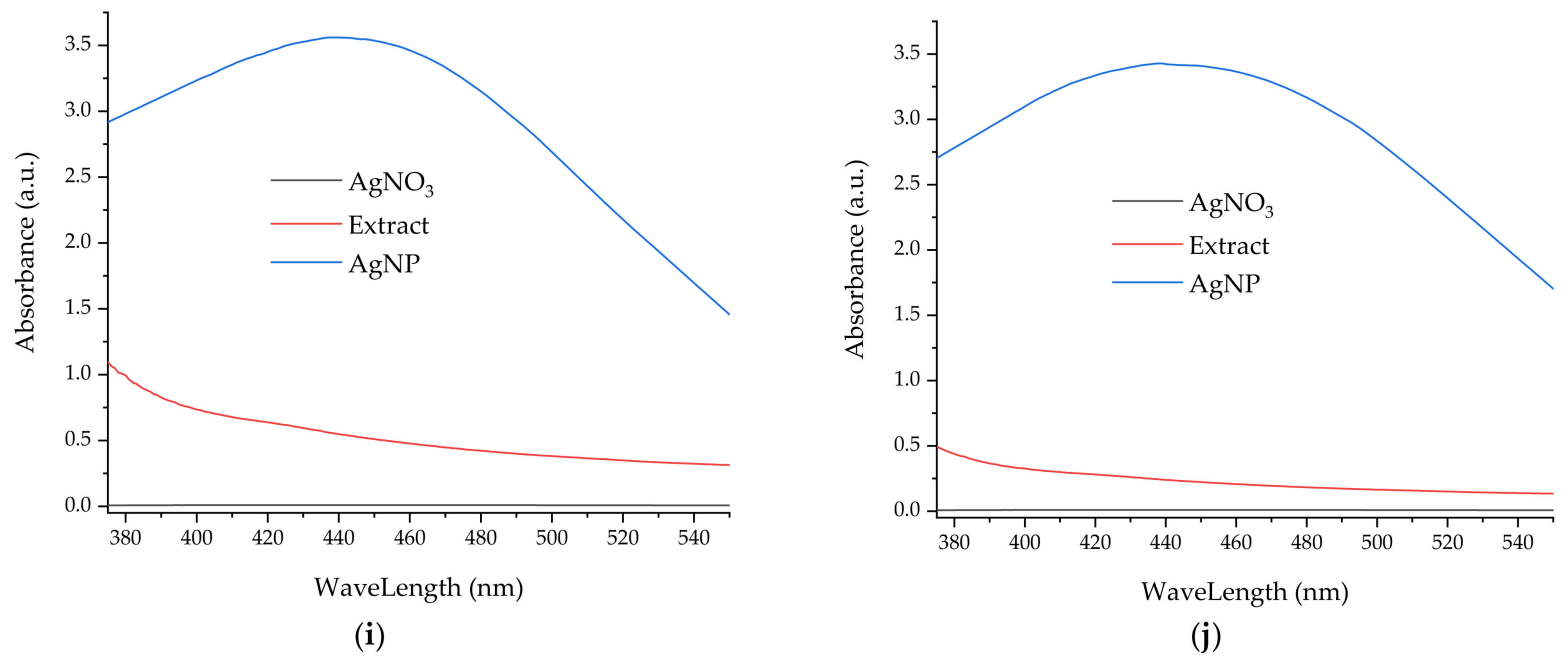
Confirmation of phytosynthesis of silver nanoparticles by comparison of nanoparticles UV-Vis spectra with the corresponding extracts and AgNO_3_ solution’s spectra: (**a**) sample LT5; (**b**) sample LT2.5; (**c**) sample LT2; (**d**) sample LT1.25; (**e**) sample LT1; (**f**) sample LM5; (**g**) sample LM2.5; (**h**) sample LM2; (**i**) sample LM1.25; (**j**) sample LM1.

Figure 4 presents the superimposed UV-Vis spectra for the nanoparticle formulations at the end of the stability evaluation period, compared to the corresponding extract, and with the silver nitrate solution, thus confirming the success of the phytosynthesis process.

**Figure 5:**
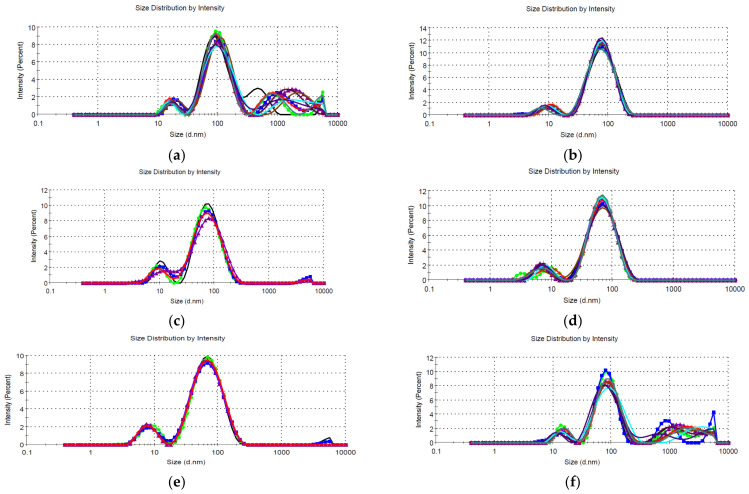

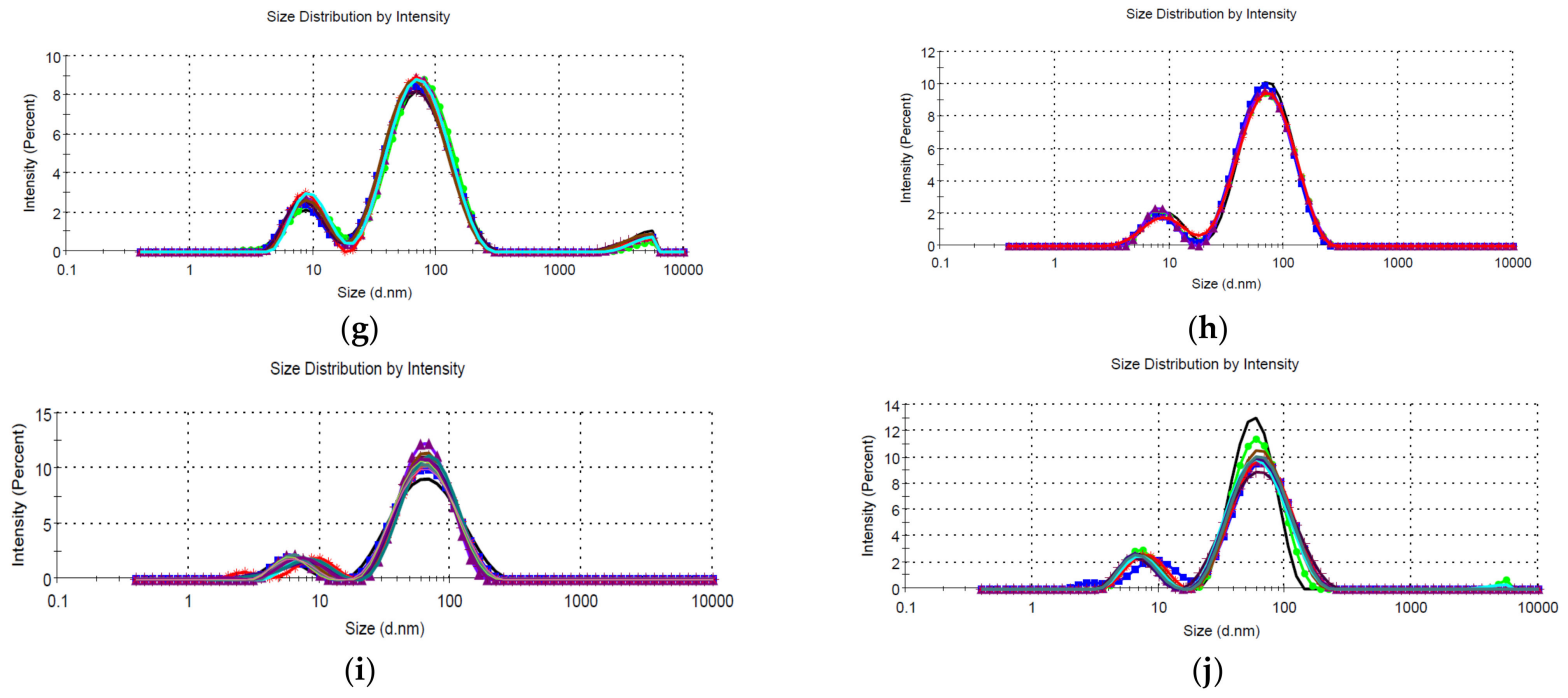
Size distribution by intensity, determined by DLS technique, for: (**a**) sample LT5; (**b**) sample LT2.5; (**c**) sample LT2; (**d**) sample LT1.25; (**e**) sample LT1; (**f**) sample LM5; (**g**) sample LM2.5; (**h**) sample LM2; (**i**) sample LM1.25; (**j**) sample LM1.

The hydrodynamic diameter of the NPs was determined using the DLS technique, by performing five/ten measurements for each sample, directly on the NPs solution, without any other treatment. The results obtained are presented in Figure 5, respectively Table 4. 

The results recorded by DLS confirms the presence of the NPs, with some larger aggregates (in the micrometer range) present in some of the samples. In evaluating the results obtained, it is important to remember that DLS determines the hydrodynamic diameter, which is usually larger than the actual particle diameter, especially for the phytosynthesized NPs [61]. What needs to be retained from the DLS results is the general trend of the NPs, with smaller dimensions being recorded for the samples developed using the lower-concentration extracts, a general trend also previously observed [39]. Additionally, it can be observed that the use of microwave-assisted extracts leads to nanoparticles having smaller diameters according to DLS measurements.

The value of the polydispersity index (PdI), with most of the samples having PdI < 0.5, could lead to the conclusion of practically monodisperse samples [39,62]. A particular case is represented by samples LT1.25, LM2, LM1.25, and LM1. For these samples, with PdI close to the threshold value of 0.5 (thus suggesting a polydisperse nature), multiple peaks are recorded, all with intensities above 9%, and all these peaks being in the nanometer range (thus confirming the nano nature of the particles). Additionally, by comparison with the results previously presented for NPs phytosynthesized using *E. purpurea* extracts [39], it can be observed that using temperature extraction, higher dimensions are obtained, while by comparing the results obtained in this study with the previously published ones [39], the dimensions of the NPs obtained using microwave extraction are smaller. By strictly comparing the results obtained in the present work, it can be concluded that the use of extracts obtained by the microwave-assisted method leads to smaller nanoparticles, compared with the extracts obtained by classical temperature extraction, at comparable concentrations.

**Figure 6:**
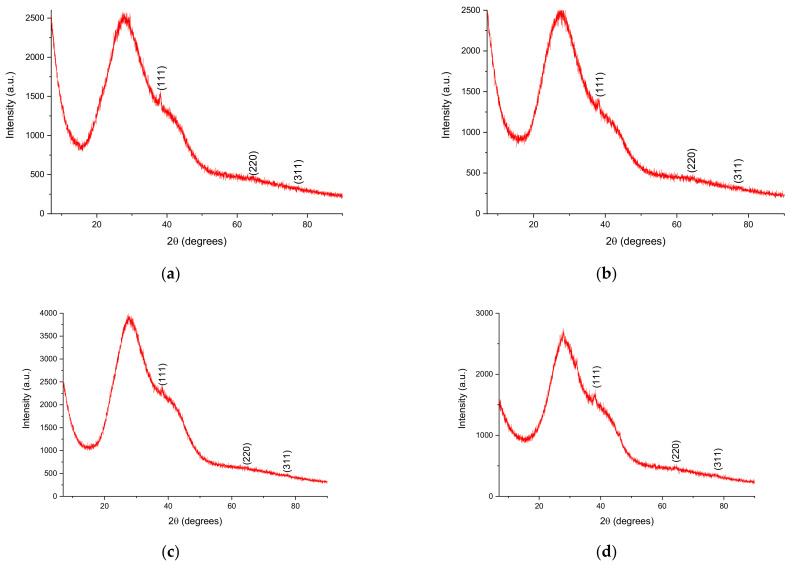

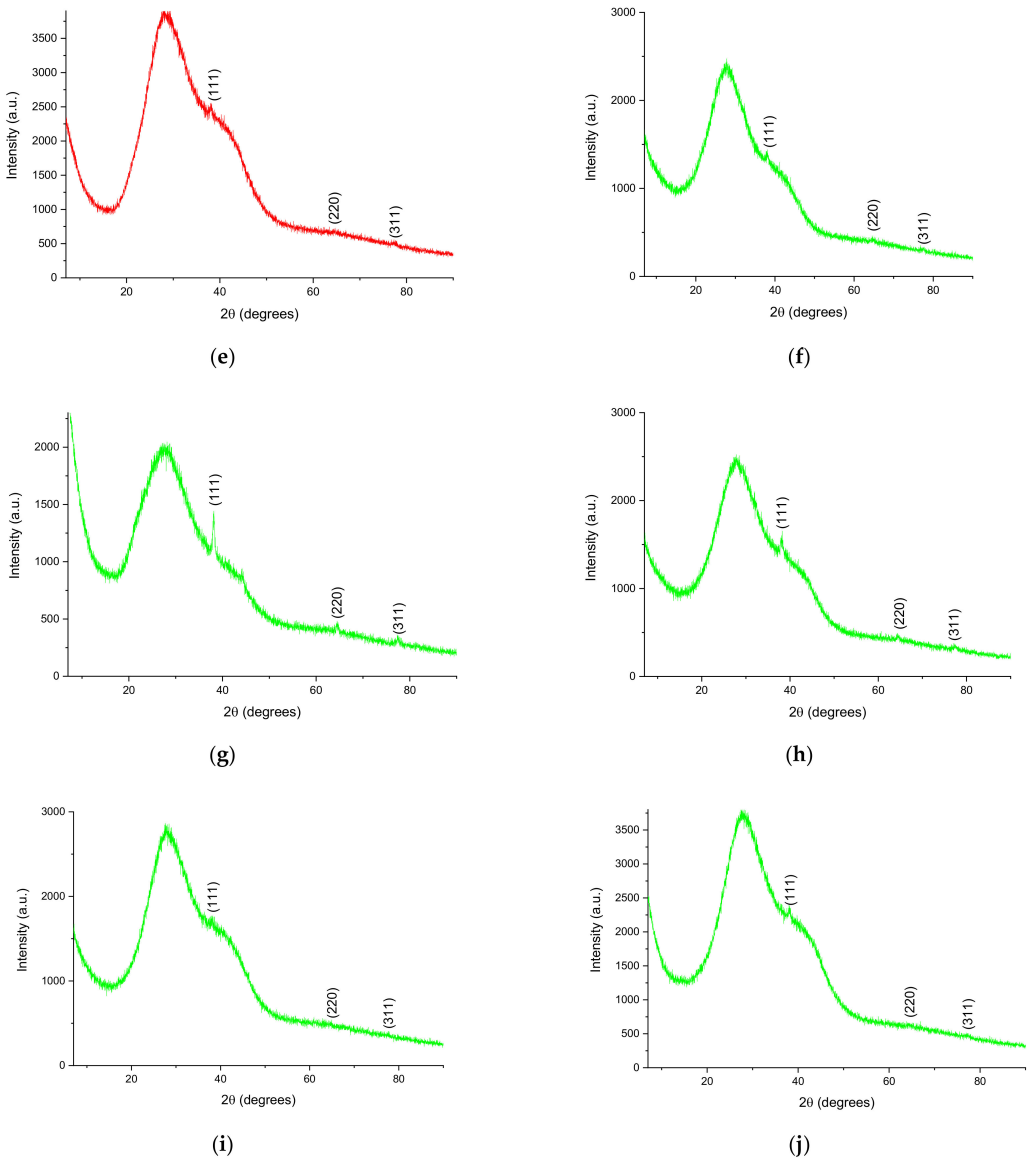
Diffractograms of the phytosynthesized nanoparticles: (**a**) sample LT5; (**b**) sample LT2.5; (**c**) sample LT2; (**d**) sample LT1.25; (**e**) sample LT1; (**f**) sample LM5; (**g**) sample LM2.5; (**h**) sample LM2; (**i**) sample LM1.25; (**j**) sample LM1.

X-ray diffraction analysis is commonly used to study the crystalline nature of different types of samples, including phytosynthesized NPs, as previously demonstrated [38,39,42]. For the analyses, the NPs solutions were centrifuged at 4000 rpms for 2 h, using a DLAB DM0408 laboratory centrifuge (DLAB Scientific Co., Ltd., Riverside, CA, USA), followed by the deposition of the obtained precipitate on the glass substrate of the equipment. The obtained diffractograms are presented in Figure 6, while XRD data (peak position and crystallite size) are presented in Table 5.

By evaluating the obtained results, it can be observed that the samples show three diffraction maxima, at 38°, 64°, and 77° (2θ, exact position presented in Table 5), corresponding to the diffraction planes (111), (220), respectively (311); the phase can be identified as Ag, in cubic crystalline system (ICDD PDF. No. 01-087-0719). 

By comparison with the results obtained for *E. purpurea* extracts, it can be observed that, for lower concentration of extracts, *L. cardiaca* leads to the development of smaller nanoparticles, with the lowest recorded crystallite size (calculated using Equation (1)) of 2.39 nm (for LM1, but values under 3 nm were also recorded for LT1.25 and LT1), compared with the crystallite sizes of 3.31, 3.40, respectively 3.15 for the NPs using the same *E. purpurea* extracts concentration and extraction methods [39].

An attempt to evaluate the correlations between the total phenolics content and the NPs crystallite size was performed using NCSS 2023 Data Analysis and Graphics software v.23.0.1 (NCSS, LLC, Kaysville, UT, USA). The Pearson coefficients (scatter graphs not shown) [63] reveal a moderate to strong correlation of total phenolic content and NPs crystallite size for the LT series (r = 0.6918) and a strong positive correlation for the LM series (r = 0.8934). This was to be expected, as for the LM series, there can be observed a linear decrease of the crystallite size with the decrease in extract concentration, while for the LT series, the highest crystallite size was observed for the 2.5 mg/mL concentration. As suggested by these results, the total phenolics content strongly influences the phytosynthesis process when applying the microwave-obtained extracts, and a weaker influence when using the temperature extraction. This could be explained by the longer extraction time of the classic method, which could lead to the extraction of other phytoconstituents playing a role in this process. Most probably, the influence of some of these phytocomponents decreases at a lower concentration (as at the lowest concentration used, 1 mg/mL, the NPs crystallite size recorded was lower for LM, compared with LT).

**Figure 7:**
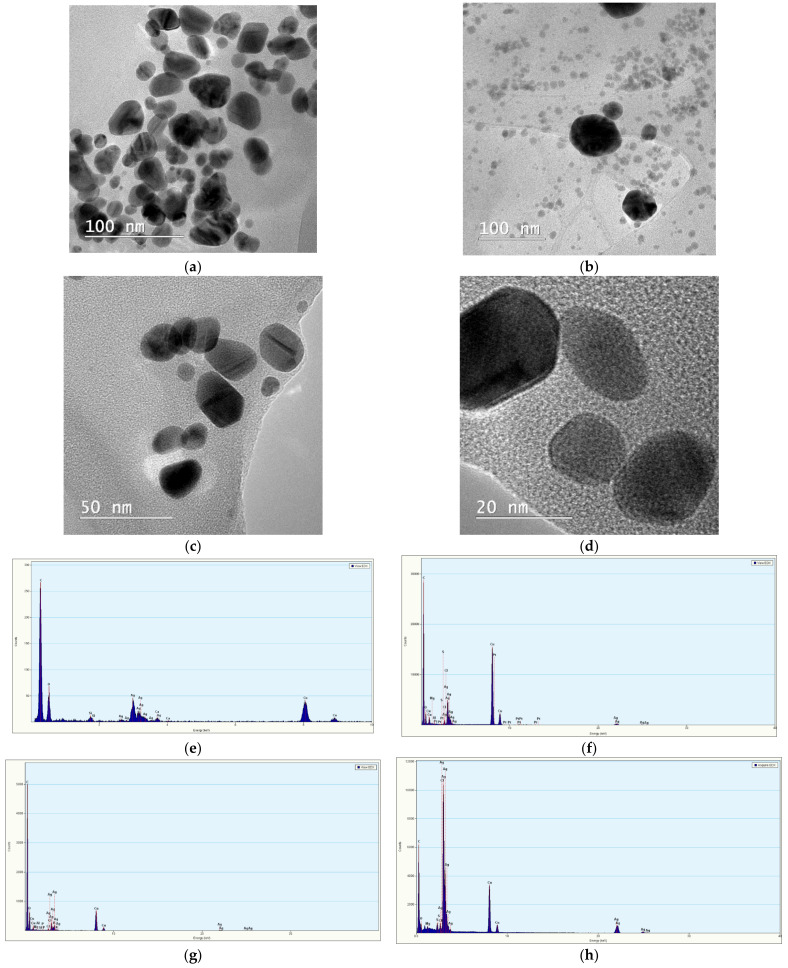

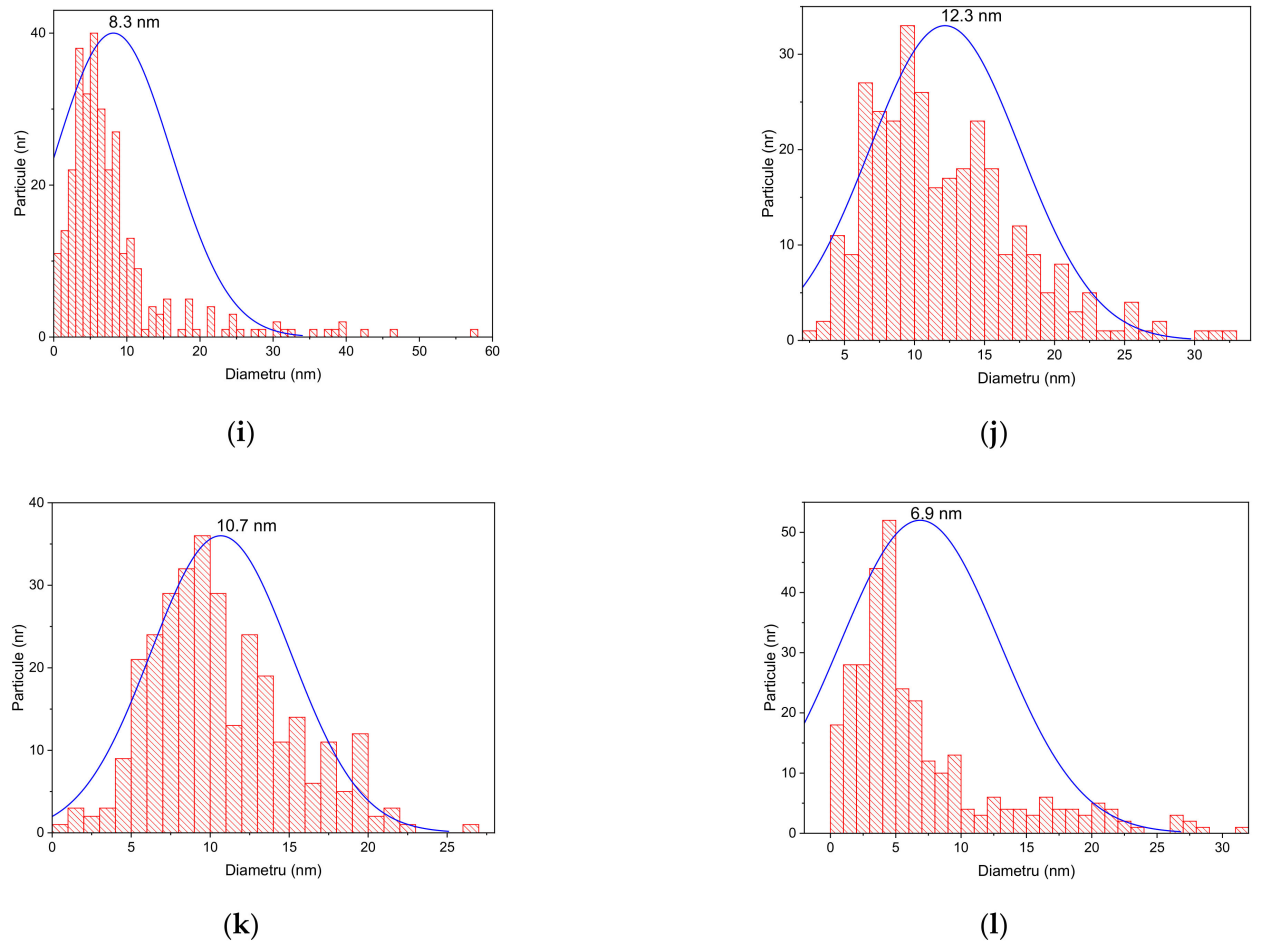
TEM images of phytosynthesized nanoparticles: (**a**) sample LT5; (**b**) sample LT2; (**c**) sample LM5; (**d**) sample LM2; EDX spectra obtained for the NPs solutions: (**e**) sample LT5; (**f**) sample LT2; (**g**) sample LM5; (**h**) sample LM2; size distribution of the NPs determined from the measurement of over 150 particles: (**i**) sample LT5; (**j**) sample LT2; (**k**) sample LM5; (**l**) sample LM2.

In order to obtain information regarding the morphologies of the samples, as well as for the visual observation of the NPs dimensions, two samples were selected for each set of NPs (samples obtained using extracts with the concentrations of 5 mg/mL, and 2 mg/mL) for TEM analysis, after the stability evaluation period (9 months). A drop of NPs solution, dispersed in water (1:10 ratio), was placed on the TEM copper grid and dried. The results are presented in Figure 7, the size distribution being evaluated from the measurement of over 150 NPs from TEM images using ImageJ image analysis software (v. 1.53s, National Institutes of Health, Bethesda, MD, USA).

TEM images present spherical/semi-spherical morphologies, with particle dimensions under 15 nm, as well as the tendency to form nanoparticles with smaller sizes for lower extracts concentration. Additionally, at the highest concentrations, the obtained dimensions are lower for the LT5, compared with LM5, while for lower concentrations, the NPs dimensions are smaller for LM2, compared with LT2 (in very good concordance with the XRD data). The EDX spectra confirm in all cases the synthesis of silver nanoparticles, the additional elements present in the spectra being due either to the TEM analysis grids (in the case of copper) or to the elements present in the extracts (in the case of the other identified elements). 

Following the analytical results, a comparison of the obtained results can also be made with literature data presenting synthesis of AgNPs using *L. japonicus* extract [35]. Thus, Im et al. characterized their NPs using UV-Vis spectrometry and TEM. The UV-Vis spectra presented by the authors showed specific peaks in the range 424–428 nm. In this respect, our results (with the presence of some peaks, for the lower concentration extracts at 418, 419, or 420 nm, as presented in Table 3), would suggest smaller dimensions nanoparticles, although at higher extract concentration, the peaks observed in the present study are located at higher wavelengths (up to 433 nm). Their TEM results (13 ± 4.2 nm) are also comparable with the ones obtained in the present study for the samples analyzed by TEM after 9 months (Figure 7).

### 3.3. Biological Properties Evaluation

**Figure 8:**
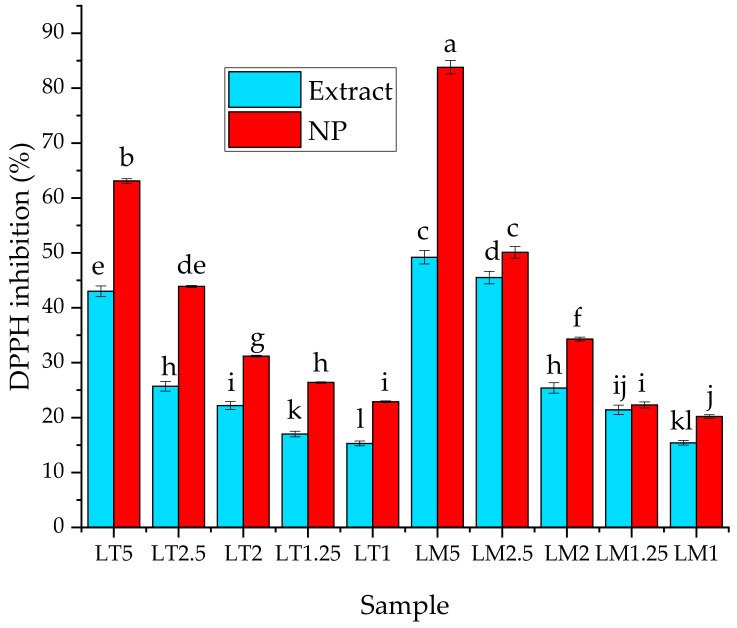
Antioxidant activity (expressed as DPPH inhibition—%) for the phytosynthesized nanoparticles and corresponding extracts. Values represent the mean ± SE, *n* = 5 per treatment group; values without a common superscript letter differ statistically (*p* < 0.05) as analyzed by one-way ANOVA and the TUKEY test.

The results regarding the determination of antioxidant activity are presented in Figure 8. 

From the results of the antioxidant assay, a statistically significant increase in the antioxidant potential upon phytosynthesis can be observed, compared with the parent extract, as also observed in our previous works [38,39,42]. By comparing the statistical significance of the recorded differences, it can be observed that, for the 1.25 and 1 mg/mL concentrations, the antioxidant potential of LT series is higher than that of the LM series, while for the other concentrations, the antioxidant potential is higher for the LM series. By comparing the two types of extracts, it can be observed that, at similar concentrations, the LM series has a stronger antioxidant potential, compared with the LT series, although the differences are not always statistically significant.

When dealing with antioxidant potential, several authors found a direct correlation between the total phenolics (and, in some extent, the total flavonoids) content and the antioxidant potential [55,64,65,66], some even for *L. cardiaca* extracts [52,67]. However, other authors found no statistic correlation between the total phenolics content and the DPPH antioxidant potential [68], some even in the study of *L. cardiaca* extracts with the same geographical origin as in our study [53]. In the present work, the antioxidant potential seems to be correlated with the flavonoids content (as both the phenolics acids determined and the total phenolics have higher values for Lt, while the antioxidant potential is significantly higher for Lmw).

**Figure 9:**
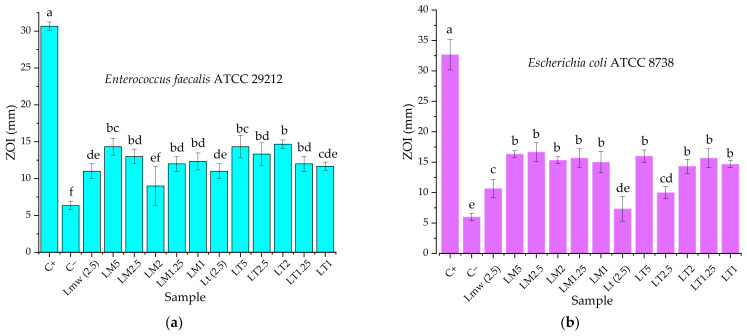
Antimicrobial activity of the tested samples against: (**a**) *Enterococcus faecalis* ATCC 29212; (**b**) *Escherichia coli* ATCC 8738. C+—positive control (as described in the Methods section); C-—negative control (water); L_mw_ (2.5)—microwave-assisted extract at 2.5 mg/mL; L_t_ (2.5)—classical temperature extract at 2.5 mg/mL. Values represent the mean ± SE, *n* = 3 per treatment group; values without a common superscript letter differ statistically (*p* < 0.05) as analyzed by one-way ANOVA and the TUKEY test.

**Figure 10:**
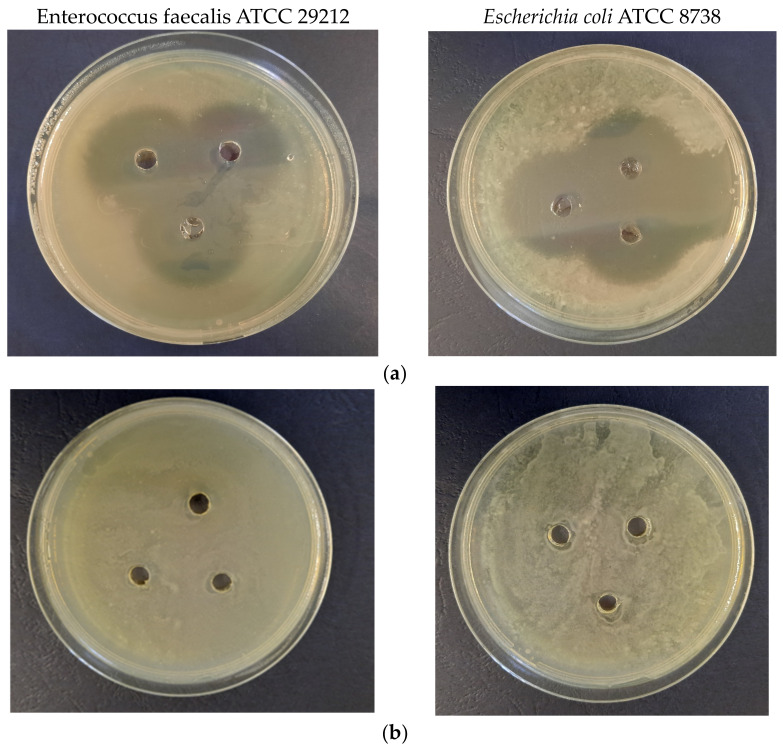

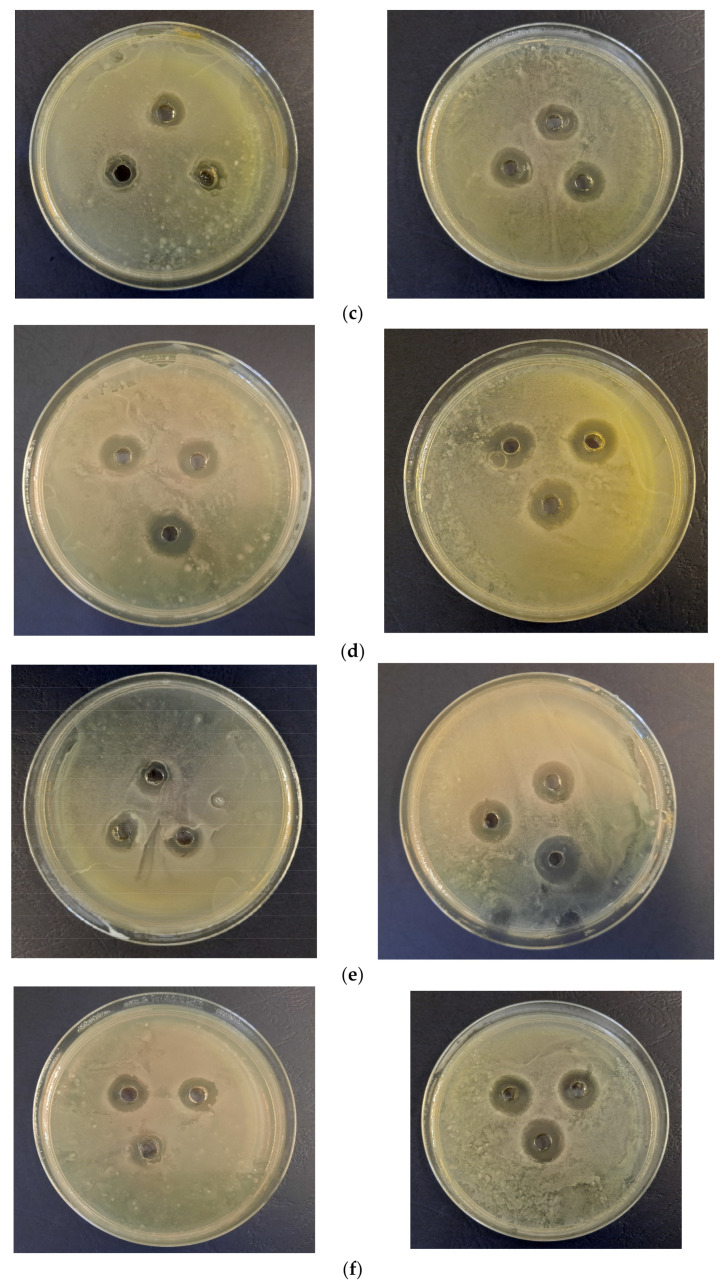
Aspect of the antimicrobial experiments (left—*Enterococcus faecalis* ATCC 29212, right—*Escherichia coli* ATCC 8738): (**a**) positive control; (**b**) negative control (as described in the Methods section); (**c**) LT1; (**d**) LT5; (**e**) LM1; (**f**) LM5.

The results of the antimicrobial properties evaluation are presented in Figure 9, while some representative images of the Petri dishes are presented in Figure 10.

Results presented in Figure 9 reveal good antimicrobial activity at all tested concentrations (with two exceptions) against both Gram-negative and Gram-positive bacteria (no significant difference being observed), with a significant increase of the activity, comparing both with the negative control, and to the secondary control. The secondary control used for the experiments consisted of the two extracts, at 2.5 mg/mL concentration (a concentration equivalent with the highest extract content that can be found in samples NPs solutions, LT5, respectively, LM5). This approach allowed us to evaluate the influence of the extract matrix on the final antimicrobial properties of the NPs. Although the differences were not statistically significant, the highest antimicrobial properties (expressed as zones of inhibition, in mm) were recorded for samples LM5 and LT2 (against the Gram-negative line), respectively, LM2.5 and LT5 (against the Gram-positive line). Additionally, as it can be seen from the comparison Lmw (2.5) vs. LM5, respectively, Lt (2.5) vs. LT5, the phytosynthesis of AgNPs enhanced the antimicrobial properties of the two extracts.

Unlike the previously presented results [39], no significant differences can be found between the NPs solution in terms of antimicrobial potential, the samples demonstrating good antimicrobial potential at all tested concentrations. 

## 4. Conclusions

The present work reports, for the first time in the literature, the phytosynthesis of silver nanoparticles using *Leonurus cardiaca* L. aerial parts extracts. The work also presents the influence of the extraction methods (classical temperature extraction and microwave-assisted extraction) and of the extract concentration on the morphological and biological properties of the NPs (established by determining the antioxidant potential and the antimicrobial properties against *Enterococcus faecalis* and *Escherichia coli*). 

The results obtained support the phytosynthesis of silver nanoparticles using *L. cardiaca* extracts, the lowest nanoparticles dimensions being obtained using the extract developed using a microwave-assisted method at a concentration of 1 mg/mL (dimensions being confirmed by UV-Vis determinations, determination of crystallite size by XRD and by DLS measurements). 

The AgNPs solutions possesses an enhanced antioxidant potential compared with the parent extracts, determined by the DPPH assay; all tested experimental variants had significant antimicrobial properties (against both Gram-negative and Gram-positive lines). At the same time, the differences between the dimensions of the nanoparticles did not strongly influence the final antimicrobial properties (as the differences recorded are not statistically significant). The antioxidant potential of the NPs was statistically influenced by the extract concentration used, being also influenced, in a smaller extent, by the NPs sizes.

The study demonstrates that the motherwort extracts are able to phytosynthesize silver nanoparticles with significant biological activities, thus allowing the proposal of nanotechnological approaches for future biomedical applications.

## Figures and Tables

**Table 1:** Encoding of the phytosynthesized nanoparticles samples.

Extract Used	Extract Concentration (mg/mL)	NP Encoding
Classical temperature extract (L_t_)	5.00	LT5
	2.50	LT2.5
	2.00	LT2
	1.25	LT1.25
	1.00	LT1
Microwave-assisted obtained extract (L_mw_)	5.00	LM5
	2.50	LM2.5
	2.00	LM2
	1.25	LM1.25
	1.00	LM1

**Table 2:** The total phenolics content (TPC) and total flavonoids (TF) determined in the analyzed extracts, respectively HPLC results ^1^.

Extract/Parameter	L_t_	L_mw_
TPC (μg GAE/g dry extract)	537.75 ± 15.44 ^a^	422.13 ± 14.15 ^b^
TF (mg RE/g dry extract)	40.80 ± 0.29 ^a^	40.91 ± 0.17 ^a^
Gallic acid (mg/g)	0.518 ± 0.026 ^a^	0.284 ± 0.014 ^b^
Chlorogenic acid (mg/g)	0.202 ± 0.007 ^a^	0.197 ± 0.007 ^a^
Coumaric acid (mg/g)	0.537 ± 0.02 ^a^	0.053 ± 0.002 ^b^
Caffeic acid (mg/g)	0.459 ± 0.019 ^b^	0.515 ± 0.021 ^a^
Hyperoside (mg/g)	0.214 ± 0.009 ^a^	0.186 ± 0.006 ^b^
Rutin (mg/g)	1.62 ± 0.065 ^a^	1.02 ± 0.031 ^b^
Naringin (mg/g)	1.78 ± 0.054 ^b^	2.27 ± 0.091 ^a^
Naringenin (mg/g)	0.273 ± 0.01 ^b^	0.322 ± 0.011 ^a^
Catechin (mg/g)	6.33 ± 0.312 ^b^	58.3 ± 2.74 ^a^
Delphinidin (mg/g)	24.5 ± 0.915 ^a^	24.8 ± 0.88 ^a^
Malvidin (mg/g)	N.D.	1.48 ± 0.061
Epicatechin (mg/g)	N.D.	N.D.
Genistein (mg/g)	N.D.	N.D.

^1^ Values represent the mean of five determinations (for TPC and TF), respectively three determinations (for HPLC results) ± SE; values in the same row without a common superscript letter differ statistically (*p* < 0.05) as analyzed by one-way ANOVA and the TUKEY test; N.D.—not detected.

**Table 3:** Evaluation of particle formation using *L. cardiaca* extracts as a function of reaction time (P_max_—characteristic peak maximum position, D_NP_—evaluation of nanoparticle sizes using characteristic maximum position).

Time	L_t_ Samples										L_mw_ Samples									
	LT5		LT2.5		LT2		LT1.25		LT1		LM5		LM2.5		LM2		LM1.25		LM1	
	P_max_ (nm)	D_NP_ (nm)	P_max_ (nm)	D_NP_ (nm)	P_max_ (nm)	P_max_ (nm)	D_NP_ (nm)	P_max_ (nm)	D_NP_ (nm)	P_max_ (nm)	P_max_ (nm)	D_NP_ (nm)	P_max_ (nm)	D_NP_ (nm)	P_max_ (nm)	D_NP_ (nm)	P_max_ (nm)	D_NP_ (nm)	P_max_ (nm)	D_NP_ (nm)
20 h	-	-	-	-	-	-	451	61	-	-	-	-	-	-	-	-	454	64	473	75
40 h	-	-	-	-	470	73	450	60	-	-	-	-	462	69	-	-	451	61	-	-
60 h	-	-	-	-	464	69	-	-	454	64	-	-	-	-	451	61	-	-	471	74
112 h	437	48	453	62	-	-	443	54	-	-	463	69	446	57	-	-	443	54	-	-
132 h	-	-	-	-	455	64	-	-	436	48	-	-	-	-	450	60	-	-	445	56
136 h	434	47	449	59	-	-	443	54	-	-	453	62	445	56	-	-	439	49	-	-
156 h	-	-	-	-	451	61	-	-	435	47	-	-	-	-	440	50	-	-	443	54
184 h	427	41	441	51	-	-	442	53	-	-	446	57	444	55	-	-	438	49	-	-
204 h	-	-	-	-	443	54	-	-	434	47	-	-	-	-	436	48	-	-	438	49
9 months	431	42	427	41	425	41	433	45	419	31	428	42	428	42	420	32	433	45	418	31

**Table 4:** Results obtained for the measurement of the particle size by the DLS method. In the table: Px—maximum (peak) X, SPx—standard deviation associated with maximum X, %Px—% intensity Px, PdI—polydispersity index.

Sample	P1	SP1	% P1	P2	SP2	% P2	P3	SP3	% P3	Average	PdI	DLS Observations
LT5	103.1	49.60	71.3	468.9	160.3	19.8	17.41	3.612	6.3	123.5	0.380	Bimodal, reproducible
LT2.5	87.46	40.80	92.7	9.756	3.605	7.3				58.21	0.302	Bimodal, reproducible
LT2	82.68	44.77	87.2	10.11	3.314	11.2	4528	863.5	1.6	46.87	0.464	Bimodal, reproducible, polydisperse, contains large aggregates
LT1.25	72.26	34.57	90.7	6.757	1.873	9.3	-	-	-	43.87	0.443	Bimodal, reproducible
LT1	73.90	34.95	86	8.532	2.987	12	4839	705.7	2	43.49	0.467	Bimodal, reproducible, polydisperse, contains large aggregates
LM5	86.54	31.31	65.8	1273	563.1	17.9	4276	976.2	8.6	87.52	0.555	Bimodal, reproducible, contains large aggregates
LM2.5	80.22	40.70	80.5	10.21	3.444	16.9	4508	876.3	2.7	39.35	0.552	Bimodal, reproducible, polydisperse, contains large aggregates
LM2	77.95	39.63	87.5	9.536	3.666	12.5	-	-	-	41.37	0.492	Bimodal, reproducible
LM1.25	72.05	35.55	89.6	6.531	1.929	10.4	-	-	-	39.86	0.487	Bimodal, reproducible, polydisperse
LM1	69.65	32.69	86.5	7.178	2.031	13.5	-	-	-	36.28	0.486	Bimodal, reproducible, polydisperse

**Table 5:** The position of the diffraction maxima and the crystallite size for the analyzed samples, calculated according to Equation (1).

Sample	(111) Peak Position (Degrees)	(220) Peak Position (Degrees)	(311) Peak Position (Degrees)	FWHM (Degrees) ^1^	Crystallite Size (nm) ^1^
LT5	37.86	64.46	77.48	2.108	4.16
LT2.5	38.28	64.67	77.12	2.081	4.22
LT2	38.28	64.75	77.55	2.107	4.17
LT1.25	38.28	64.60	77.48	3.317	2.65
LT1	38.21	64.59	77.19	3.387	2.59
LM5	38.14	64.39	77.12	1.609	5.45
LM2.5	38.07	64.56	77.38	1.965	4.47
LM2	38.07	64.42	77.55	2.249	3.90
LM1.25	38.28	65.53	76.55	2.321	3.78
LM1	37.99	64.75	77.62	3.673	2.39

^1^ Data for (111) diffraction plane.

## Data Availability

The data presented in this study are available on request from the corresponding author.
